# Supplementary material for: Content-rich biological network constructed by mining PubMed abstracts
Source: BMC Bioinformatics. 2004 Oct 8;5:147. doi: 10.1186/1471-2105-5-147 (PMC528731; doi:10.1186/1471-2105-5-147)
Supplement: Additional File 2 — The original results of the above study (non-essential files are deleted to keep the file size under the limit set by BMC bioinformatics). [file 1471-2105-5-147-S2.bz2 › chilibotAdditionalFile2/dip05/767ID9732274E18156/html/DNAK_PNP.html]

 


 **DNAK** and **PNP** 
  
Found 8 abstracts in PubMed, retrieved 05.  
 

 What does Google say? 
 PDF only 
| .edu only 

---

**Interactive relationship** (e.g. stimulation, inhibition, etc)

**Inhibitory relationship**- Injection of  **pNP**  hsp 70  [ **DNAK** ]  complexes although generating good primary responses, resulted in very much decreased proliferative responses to the hsp 70  [ **DNAK** ] .  Ref: 8881747 Immunology, 1996
**Neutral relationship**- Incubation of isolated axonemes in ATP, but not AMP or AMP  **PNP** , caused significant release of the previously bound HSP70  [ **DNAK** ]  as is characteristic of complexed HSP70s.  Ref: 8586665 J Cell Sci, 1995

**Non-interactive relationship** (e.g. studied together, co-existance, homology, etc.)

- N terminal sequencing of these proteins revealed the presence in the RNase E complex not only of polynucleotide phosphorylase  [ **PNP** ]  but also of  **DnaK** , RNA helicase, and enolase.  Ref: 8632981 Proc Natl Acad Sci U S A, 1996
- We have examined the immunogenicity of complexes formed by non covalent association of a synthetic peptide corresponding to influenza A virus nucleoprotein, residues 206 229  **pNP**  and Mycobacterium tuberculosis heat shock protein 70  [ **DNAK** ]  hsp 70  [ **DNAK** ] .  Ref: 8881747 Immunology, 1996
